# Influence of Die Land Length on the Maximum Extrusion Force and Dry Ice Pellets Density in Ram Extrusion Process

**DOI:** 10.3390/ma16124281

**Published:** 2023-06-09

**Authors:** Jan Górecki, Wiktor Łykowski

**Affiliations:** Faculty of Mechanical Engineering, Institute of Machine Design, Poznan University of Technology, 60-965 Poznań, Poland

**Keywords:** densification, extrusion, single-cavity die, die land length, dry ice, carbon dioxide

## Abstract

The management of waste materials, particularly non-biodegradable substances such as plastics and composites, is an increasingly pressing issue. Energy efficiency in industrial processes is crucial throughout their life cycle, including the handling of materials such as carbon dioxide (CO_2_), which has a significant environmental impact. This study focuses on the conversion of solid CO_2_ into pellets using ram extrusion, a widely used technique. The length of the die land (DL) in this process plays a critical role in determining the maximum extrusion force and the density of dry ice pellets. However, the influence of DL length on the characteristics of dry ice snow, known as compressed carbon dioxide (CCD), remains understudied. To address this research gap, the authors conducted experimental trials using a customized ram extrusion setup, varying the DL length while keeping the other parameters constant. The results demonstrate a substantial correlation between DL length and both the maximum extrusion force and dry ice pellets density. Increasing the DL length leads to a decreased extrusion force and optimized pellet density. These findings provide valuable insights for optimizing the ram extrusion process of dry ice pellets and improving waste management, energy efficiency, and product quality in industries utilizing this technique.

## 1. Introduction

Management of waste materials is becoming a topical issue for researchers and a growing challenge for civilisation. This concerns in particular the materials that are not inert to the natural environment, such as plastics [1,2] and composites [3,4]. In regards to biodegradable materials, traditional methods such as composting [5] may turn out to be more beneficial than other processing methods [6]. Hence, particular attention should be paid to the energy efficiency of all the industrial processes, to which a secondary material will be subjected throughout its entire life cycle [7].

**Figure 1:**
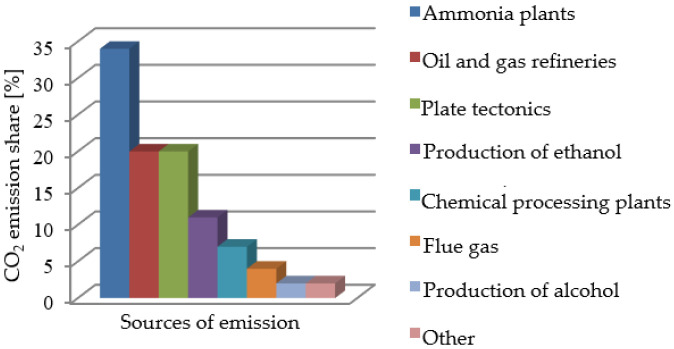
Carbon dioxide emission sources [10,11].

Waste materials that have a considerable effect on the natural environment include carbon dioxide [8]. As can be seen in Figure 1, waste carbon dioxide is generated in various industrial processes and also during the processing of different energy sources, such as fuels. Taking into account a considerable percentage of processes related to the production of ammonia compounds, manufacturers recover carbon dioxide in order to reuse it in other production/industrial processes or deliver it to interested third parties, as the case may be [9].

Carbon dioxide has many uses in its solid state. Its temperature-related physical characteristics (i.e., 194.2 K temperature in the solid form and sublimation at normal ambient temperatures) [12,13] are desired in various applications including food refrigeration [14], disinfection of liquids [15], and abrasive cleaning of surfaces [16,17].

**Figure 2:**
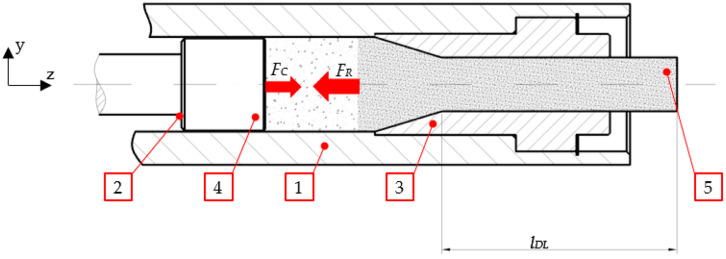
The main part of the piston-type pelletizer. 1—compaction chamber, 2—piston, 3—single channel die, 4—dry ice before compression, 5—compressed dry ice, *l_DL_*—length of DL section.

In order to be used in these applications, carbon dioxide must be turned into pellets. Ram extruders are the machines used for this purpose. The ram extruder’s working system has been extensively described in the literature, for example in [18] where the reader can find details supplementing the following description that has been purposefully limited to the elements related to the research subject (Figure 2).

**Figure 3:**
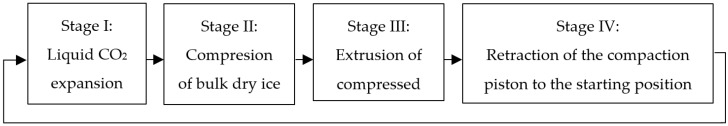
Sequence of dry ice production stages.

The process of extruding dry ice pellets is carried out in the sequence of stages presented in Figure 3. In the first stage, liquid carbon dioxide is expanded to obtain a solid material inside the densification chamber. According to Wałęsa et al. (2022), in the second phase, the ram presses the dry ice particles until the force exerted on the ram (*F*_C_) equals the resistance force (*F_R_*), depending on the geometry of the die that the material is pushed through [19]. While moving through the converging portion of the die cavity, the material undergoes plastic deformation. Its transverse dimension is determined by the geometry of the die cavity through which it is pushed. In the next phase, the material is moved through the die land section (DL) in the direction normal to the *Z* axis. The transversal shape of this portion of the die cavity approximates the shape of the pellets that leave the die. After the completion of the densification process, the compaction piston is withdrawn to its initial position.

**Figure 4:**
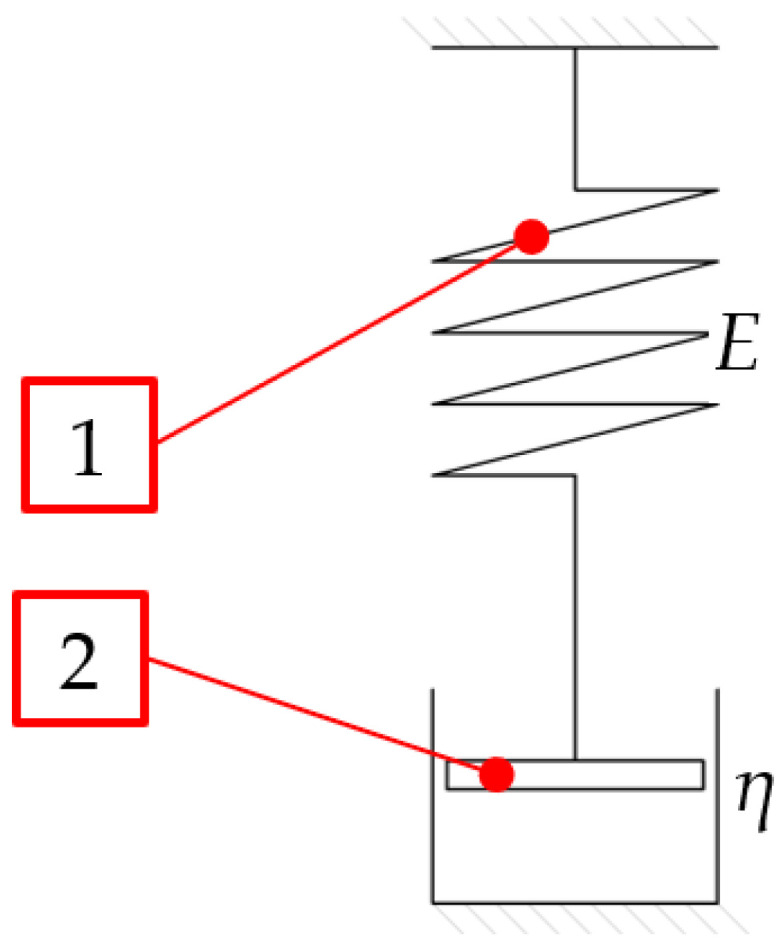
Schematic diagram of Maxwell model, 1—purely elastic body, 2—purely viscous body.

The DL portion is introduced due to the high plastic deformation of the material during compaction. As a result, the stress tensor (*σ*_0_) may substantially exceed the material’s cohesion. Thus, we can adopt a non-zero absolute value of the deformation tensor (*ε*). With constant transverse dimensions over the whole DL length, in axes other than the *Z* axis, ε equals zero. If the transversal dimensions of the pressed material are constant, the phenomenon of stress relaxation comes into play. This phenomenon is described, for example, with the Maxwell model for linear viscoelastic response. It combines a purely elastic body with an ideally viscous body, represented by a spring and a damper (as shown in Figure 4). The above system may be described by Equation (1):

(1) $$\sigma \left(t\right)=\sigma_{0}\cdot e^{\left(\frac{-t}{T_{R}}\right)},$$

where *σ*_0_ represents initial stress and *T_R_* represents total relaxation time.

The variable of the initial stress (*σ_0_*) resulting from the material relaxation phenomenon is time-dependent. In this case, *t* depends on the DL length (*l_DL_*) and the speed of movement of the material (*v_DL_*_,_) (as shown in Figure 2). The maximum value of *t* is calculated as follows:

(2) $$t_{DL}=\frac{l_{DL}}{v_{DL}}$$

According to Biszczanik et al. (2021), on the way through the convergent section, the density of pressed dry ice does not change considerably [20]. Hence, the approximate value of *v_DL_* may be determined assuming the incompressibility of the material over the convergent section. It depends on the initial speed of the material (*v^in^*) and the *S^in^* to *S^out^* ratio (convergent section inlet and outlet cross-sectional areas). Now Equation (2) may be rewritten as follows:

(3) $$t_{DL}=l_{DL}\cdot \frac{S^{in}}{v^{in}\cdot S^{out}}.$$

Substituting *t_DL_* in Equation (1) yields:

(4) $$\sigma \left(t_{DL}\right)=\sigma_{0}\cdot e^{\left(-\frac{l_{DL}\cdot S^{in}}{T_{R}{\cdot S}^{out}}\right)}.$$

According to Biszczanik et al. (2021), dry ice pellets show elastic behaviour [20]. Therefore, in accordance with Hook’s model, relaxation is deemed to decrease the material deformation of *ε* after leaving the die. Note that the value of *ε* has a direct bearing on the extrudate density (*ρ*), a parameter indicated as one of the dry ice pellets quality indicators [21].

However, the Maxwell model does not take into account the material sublimation phenomenon. Note that the sublimation rate may vary considerably depending on the friction between the DL section’s sides and the pressed material.

Reports from the investigation of the influence of the DL length and surface condition on the end product density and process forces can be found in the literature [22]. However, considering the peculiar properties of dry ice snow (a.k.a. CCD) the authors have noted a gap in knowledge to be filled by the publication of the outcome of the experimental research presented herein. This will allow the determination of the inaccuracy of the Maxwell model’s representation of the process in question due to reasons including sublimation.

## 2. Materials and Methods

### 2.1. Materials

#### 2.1.1. Dry Ice Powder

Dry ice powder is obtained by expanding the atmospheric pressure of liquid carbon dioxide stored in a tight container at −18 degrees Celsius and 20 bar pressure. This rapid expansion to the atmospheric pressure results in an adiabatic process and converts liquid CO_2_ into a solid form. The material crystallises in this way. The density of the obtained loose dry ice is 550 kg/m^3^ [23].

**Figure 5:**
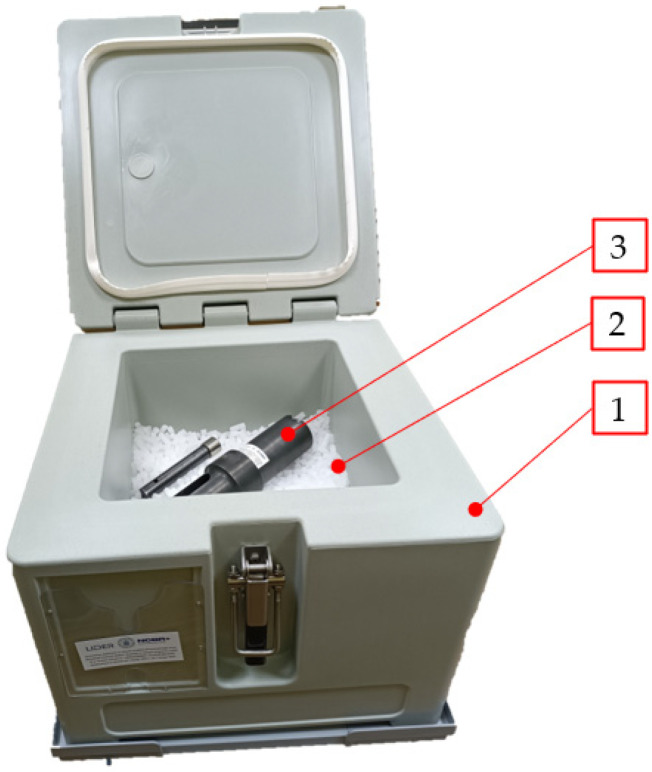
Insulated container used to store dry ice powder and condition the experimental setup components, 1—insulated container, 2—dry ice, 3—experimental setup.

For the purposes of this experimental research, loose dry ice was stored in a 30 L insulated DRICY dry ice storage container (Figure 5) manufactured by Melform (Monasterolo di Savigliano, Italy). An insulated container was used in order to reduce the sublimation rate. A second container of the same type was used to condition the experimental setup components at a temperature approximating the tested material temperature.

#### 2.1.2. Compression and Extrusion

In the process comprising the compression and extrusion phases, loose dry ice was pushed by a ram through a single-cavity die. The experimental setup was built by the authors specifically for the verification of limit stress in the dry ice extrusion process. In this research, this test setup was mounted in the grips of the MTS Insight 50 kN test frame manufactured by MTS Systems Corporation (Eden Prairie, MN, USA). The test setup is described in detail in earlier reports [24].

The measured variable was the force (*F_Z_*) exerted on the 20 mm dia. ram. A 50 kN accuracy class 0.01 strain gauge sensor was used for this measurement, supplied as standard with the MTS Insight 50 kN test frame.

The specimen used in the experiment was a 20 g portion of loose dry ice, pressed until the ram reached the pre-determined position of 1 mm above the top edge of the die. This procedure allowed us to obtain product samples of repeatable extrudate density that depended on the geometry of the tested single-cavity extrusion die.

#### 2.1.3. Dies

**Figure 6:**
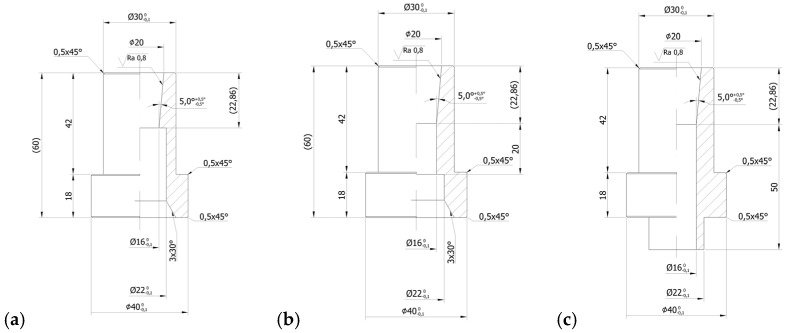
Geometrical parameters of some of the tested single-cavity dies, (**a**) zero *l_DL_*, (**b**) *l_DL_* = 20 mm, (**c**) *l_DL_* = 50 mm.

Single-cavity dies with conically converging sections were used. The converging section parameters were beyond the scope of this research and based on earlier publications. As a result, it was decided to use dies with a 5-degree convergence angle. The varying parameter of the tested die was the DL length (*l_DL_)*, which varied from 0 mm to 50 mm. The geometrical parameters of some of the tested dies are illustrated below (Figure 6).

For the purpose of the investigation subject, it was required to determine the *l_DL_* range in which the greatest changes of the limit force of *F_Z_* and *ρ* are observed. To this end, dies with the *l_DL_* increasing at 10 mm intervals (i.e., 0, 10, 20, 30, 40 and 50 mm) were used in the first stage. Next, measurements were carried out on dies with smaller *l_DL_* intervals falling in the range determined based on the previously obtained results. The details are given in Section 3 further below.

### 2.2. Method of Measurement of the Maximum Extrusion Force

**Figure 7:**
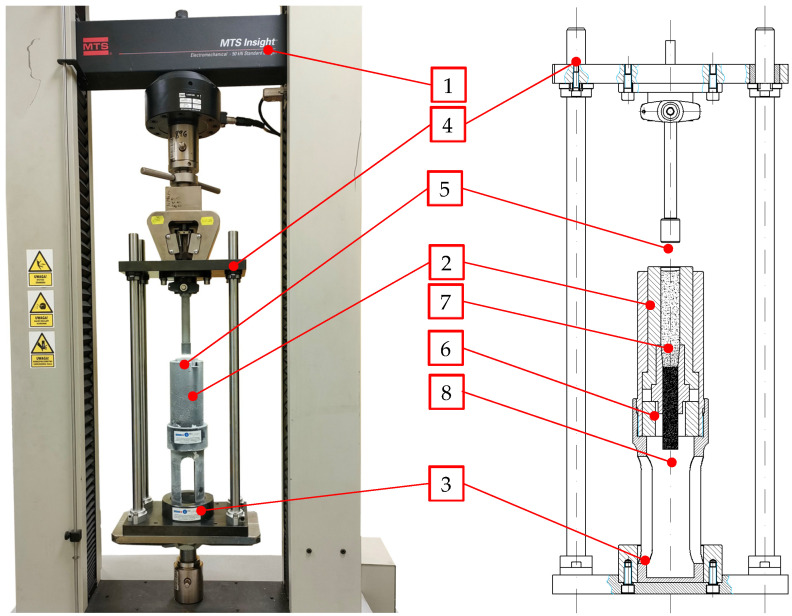
Dry ice compaction and extrusion test setup, 1—MTS Insight 50 kN test frame, 2—Upper sleeve with the compaction chamber contained inside it, 3—Lower sleeve, 4—Guide assembly, 5—Ram, 6—Single-cavity die, 7—Loose-dry ice, 8—Dry ice pellets.

The force measurement method used in this study was described by Biszczanik et al. (2023) [24]. Dry ice powder was compacted into 16 mm diameter pellets using a specially designed dry ice compression/extrusion system, including single-cavity dies. The test setup shown in Figure 7 employs the ram extrusion technique. It is composed of the MTS Insight 50 kN (1) test frame with fitted dry ice compaction setup and a computer with installed machine operation software version 4.0 that records the measurement results. The test setup is made up of several characteristic parts: guide assembly (4), ram (5) held by the test frame grips, sleeve with the compaction chamber (2) and the extrusion die placed inside it, positioned in the bottom part of the guide assembly (6). As can be seen in Figure 7, the parts, such as the ram (5) and the sleeve that contains the compaction chamber (6) and the die, are cold, as manifested by the frost on their outside surfaces.

The specific test setup elements were cooled prior to measuring in order to reduce the effect of temperature by minimising the loss of the material mass through sublimation. The parts were cooled by submersion in dry ice to obtain a temperature approximating the temperature of the test material itself. The compaction chamber, along with the die and the ram, were cooled for 30 min before the tests. Next, during the tests, these parts were re-cooled for 10 min after every three consecutive material extrusion cycles (approx. every 6 min) in order to keep the temperature of the test setup close to the dry ice temperature.

The MTS Insight 50 kN test frame equipped, with the TestWork4 software version 4.0, makes it possible to simultaneously record the extrusion force and the ram displacement. The first operation performed before the proper measurements were complete was to plug the end of the die placed in the chamber by pouring, compacting, and extruding the material several times (these results were ignored). As a result, the densified material remained in the die, which prevented the dry ice from getting through the die while filling the chamber with it. Only after cohesive pellets extruded through the die were obtained, could the proper testing start. In the course of the tests, the compaction chamber was filled with loose dry ice, the amount being 20 g pre-weighed on the Keller PCB10000-1 scale (London, UK) with 0.1 g accuracy. In the next step, the program was initialized, moving the ram at a speed of 5 mm/s into the chamber and stopping 1 mm before the die edge (to prevent its possible damage), and simultaneously recording the force values and displacements. Ten tests were carried out for each die.

The recorded maximum forces (*F_C_^max^*) are described in the groups assigned to the tested single-cavity dies.

### 2.3. Extrudate Density Measurement

**Figure 8:**
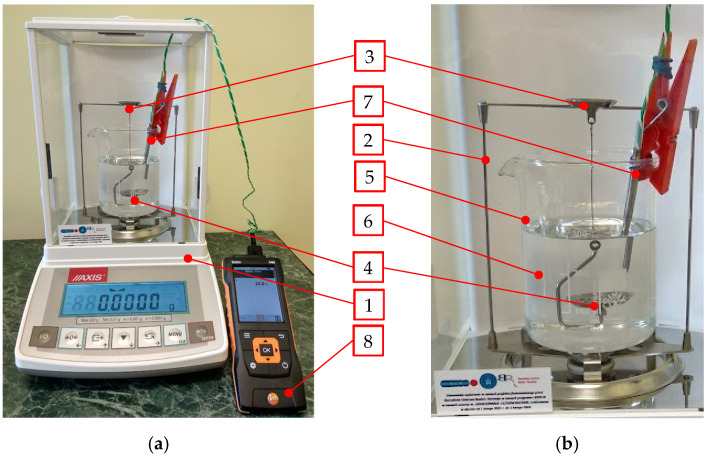
Hydrostatic extrudate density measurement: (**a**) overview, (**b**) close-up of the hydrostatic test module; 1—ACN220 balance with the draft shield, 2—hydrostatic test setup, 3—upper plate, 4—lower plate, 5—beaker, 6—test liquid, 7—K-type thermocouple, 8—Testo 440 multimeter used to measure the temperature.

The hydrostatic method was chosen considering the sublimation of the tested material and the irregular shape of the produced pellets. In this method, the dry weight of the sample (*m*_0_) is compared with *m*_1_ (the weight of the same sample), immersed in the test liquid of known density (*ρ*_TL_). The test setup is shown in Figure 8 below.

Isopropyl alcohol was used to allow for the cooling of the liquid to a very low temperature. The value of *ρ*_TL_ varies as a function of *T*_TL_, and thus the test setup was equipped with a K-type thermocouple sensor (7) and a Testo 440 multimeter (Pruszków, Poland) (8) to read the sensor output signal.

The equations derived in the previous studies [25] allowed for the determination of *ρ*_TL_ and *ρ*_CCD,_ based on the measured values of *m*_0_, *m*_1_, and *T*_TL_. The equations used to calculate *ρ*_TL_ and *ρ*_CCD_ are given below:

(5) $$\rho_{CCD}=\frac{m_{0}}{m_{0}-m_{1}}\rho_{L}\left(T_{TL}\right),$$

(6) $$\rho_{TL}\left(T_{TL}\right)={-0.947\cdot T}_{TL}+1066.$$

### 2.4. Statistical Analysis

One-way ANOVA, along with Tukey’s post hoc test, was used to assess the mean differences of *F_C_^max^* and *ρ*_CCD_ between the data groups. STATISTICA version 13.3, a program of TIBCO Software Inc. was used for the statistical analysis of the experimental data. In all, the comparison one-way test was applied with statistical significance, determined by the value of *p* below 0.05.

## 3. Results & Discussion

The statistical significance of the differences between the data in the DL0, DL10, DL20, DL30, DL40 and DL50 populations was verified by means of the Tukey post hoc test. For this purpose, the null hypotheses of ANOVA were first verified.

The Shapiro–Wilk test was applied in the first step to verify the normality of the data in the populations. In the case of *F_C_^max^*, the value of *p* varied from 0.11 to 0.80. For *ρ*_CCD_, the range was 0.16–0.62. The values obtained for each of the tested populations exceed 0.05, which confirms the normality of the data.

Next, Levene’s test was used to verify the homogeneity of data distributions. The value of *p* was 0.54 for *F_C_^max^* and 0.84 for *ρ*_CCD_, indicating the homogeneity of variance in both cases.

The analysis of variance with Tukey’s post hoc test showed statistically significant differences between the results obtained for the die with an *l_DL_* equal to zero and the other dies under analysis. The general population data are given in Table 1 and Table 2 below.

We decided to carry out additional tests for *l_DL_*s of 5 mm and 2.5 mm. In this way, it was determined that the *l_DL_* variation range should be limited to the range of 0–2.5 mm.

In the next step, the tests were carried out for dies with DLs equal to 0, 1, 1.5, 2 and 2.5 mm, respectively. The same, as in the previous assessment, Shapiro–Wilka and Levene’s tests were used to verify the ANOVA null hypotheses. This verification confirmed the normality of data in the respective populations and the homogeneity of variance. The statistical significance analysis showed that the data obtained for the dies with *l_DL_*s in the range of 0–1.5 mm are different from other populations at a statistically significant level. The key statistical data obtained for these two tests are given in Table 3 and Table 4 below.

Analysing the change of *ρ*_CCD_, as a function of *l_DL_*, we see a decrease in the variation of *ρ*_CCD_ values for *l_DL_*s greater than 1.5 mm. Therefore, we can assume that the stress resulting from the plastic deformation of the sample was reduced to a level at which its effect on the geometrical parameters of the pellets becomes ignorable.

Based on this conclusion, the minimum value of *l_DL_* for the relaxation to take place in the compacted carbon dioxide should be taken at 1.5 mm. Substituting these parameters in Equation (3), it is now possible to calculate *t_DL_*:

(7) $$t_{DL}=l_{DL}\cdot \frac{S^{in}}{v^{in}\cdot S^{out}}=0.78\;s.$$

Due to the exponential growth of stress (*σ*) calculated with Equation (1), the value of stress inside the pellets is reduced to a level at which it no longer causes any significant deformation of the extrudate.

The tests showed that *l_DL_* has a bearing on the mechanical properties of dry ice pellets and on the extrusion force used in the process. This is in line with the conclusions of the relevant studies on pelletizing of wood chips [26], straw [27], and other particulate materials [28].

In the literature, *t_R_* is described as a product of dynamic viscosity (η and E) [29]. Considering the variability of the mechanical parameters of dry ice as a function of *ρ* [20,30], the results are deemed applicable for extrudates whose density does not exceed the extrudate density determined in the experiments (i.e., 1540 kg/m^3^).

## 4. Conclusions

This experimental research showed the need for a cylindrical section in dry ice compaction and extrusion dies. It was found to have a considerable bearing on the extrudate density and extrusion force required in the process. The indicated information has also been confirmed in the case of the densification process of other particulate materials (e.g., by Brewin et al. (2008) [27]).

For the extrudate density of ca. 1540 kg/m^3^ and a ram speed of 5 mm/s, the minimum relaxation time was 0.78 s. This allowed for a reduction of the stress induced inside the pellets to a level at which it no longer caused any significant deformation of the extrudate and, consequently, no density change.

The outcome of this research could serve as the basis for further investigations of the dry ice relaxation phenomenon. The authors plan to use the Maxwell model described in this article and also the Kelvin–Voigt model to develop a material model for use in numerous studies [31,32]. Supplementing the existing lack of knowledge regarding mathematical models describing the phenomena occurring in the dry ice extrusion process, as indicated by Wójcik and Skrzat in 2018 (in the case of material extrusion in the KOBO process), can positively impact the improvement of energy efficiency in industrial processes [33].

The future objective is to advance the research and development efforts to improve the energy efficiency of industrial and geological processes used to increase the density of solid carbon dioxide [11,34].

## Figures and Tables

**Table 1:** General population data of *F_C_^max^*, kN for DL range of 0–50 mm.

DL	Min	Q25	Q50	Q75	Max	AVR
0	7.9	9.1	9.5	10.1	10.5	9.5
10	8.2	8.5	8.9	9.3	10.6	9.0
20	7.6	7.7	8.5	8.8	9.0	8.3
30	7.9	8.1	8.6	8.9	9.3	8.6
40	8.3	8.7	3.4	9.6	10.3	9.3
50	8.2	8.5	8.9	9.0	9.3	8.8

**Table 2:** General population data of *ρ*_CCD_, kg/m^3^ for DL range of 0–50 mm.

DL	Min	Q25	Q50	Q75	Max	AVR
0	1170	1220	1300	1360	1480	1310
10	1440	1460	1480	1520	1560	1490
20	1310	1410	1430	1460	1530	1440
30	1380	1450	1520	1530	1550	1490
40	1280	1320	1360	1570	1660	1430
50	1310	1400	1430	1470	1490	1420

**Table 3:** General population data of *F_C_^max^*, kN for DL range of 0–2.5 mm.

DL	Min	Q25	Q50	Q75	Max	AVR
0	7.9	9.1	9.5	10.1	10.5	9.5
0.5	7.4	7.5	7.9	8.1	8.2	7.8
1	7.5	7.7	7.8	8.6	9.6	8.6
1.5	7.8	8.3	8.5	9.3	9.4	8.7
2.0	7.8	8.3	8.6	9.0	9.4	8.6
2.5	7.8	8	9.0	9.7	9.9	8.9

**Table 4:** General population data of *ρ*_CCD_, kg/m^3^ for DL range of 0–2.5 mm.

DL	Min	Q25	Q50	Q75	Max	AVR
0	1170	1220	1300	1360	1480	1310
0.5	1310	1320	1380	1390	1500	1380
1	1310	1330	1370	1420	1440	1370
1.5	1430	1460	1490	1500	1540	1480
2.0	1450	1530	1530	1540	1560	1530
2.5	1410	1500	1530	1590	1640	1540

## Data Availability

Not applicable.
